# The p38 MAPK Regulates IL-24 Expression by Stabilization of the 3′ UTR of IL-24 mRNA

**DOI:** 10.1371/journal.pone.0008671

**Published:** 2010-01-13

**Authors:** Kristian Otkjaer, Helmut Holtmann, Tue Wenzel Kragstrup, Søren Riis Paludan, Claus Johansen, Matthias Gaestel, Knud Kragballe, Lars Iversen

**Affiliations:** 1 Department of Clinical Immunology, Aarhus University Hospital, Aarhus, Denmark; 2 Department of Dermatology, Aarhus University Hospital, Aarhus, Denmark; 3 Institute of Biochemistry, Medical School Hannover, Hannover, Germany; 4 Institute of Medical Microbiology and Immunology, Aarhus University, Aarhus, Denmark; Dana-Farber Cancer Institute, United States of America

## Abstract

**Background:**

IL-24 (melanoma differentiation-associated gene-7 (mda-7)), a member of the IL-10 cytokine family, possesses the properties of a classical cytokine as well as tumor suppressor effects. The exact role of IL-24 in the immune system has not been defined but studies have indicated a role for IL-24 in inflammatory conditions such as psoriasis. The tumor suppressor effects of IL-24 include inhibition of angiogenesis, sensitization to chemotherapy, and p38 mitogen-activated protein kinase (MAPK)-mediated apoptosis. Current knowledge on the regulation of IL-24 expression is sparse. Previous studies have suggested that mRNA stabilization is of major importance to IL-24 expression. Yet, the mechanisms responsible for the regulation of IL-24 mRNA stability remain unidentified. As p38 MAPK is known to regulate gene expression by interfering with mRNA degradation we examined the role of p38 MAPK in the regulation of IL-24 gene expression in cultured normal human keratinocytes.

**Methodology/Principal Findings:**

In the present study we show that anisomycin- and IL-1β- induced IL-24 expression is strongly dependent on p38 MAPK activation. Studies of IL-24 mRNA stability in anisomycin-treated keratinocytes reveal that the p38 MAPK inhibitor SB 202190 accelerates IL-24 mRNA decay suggesting p38 MAPK to regulate IL-24 expression by mRNA-stabilizing mechanisms. The insertion of the 3′ untranslated region (UTR) of IL-24 mRNA in a *tet-off* reporter construct induces degradation of the reporter mRNA. The observed mRNA degradation is markedly reduced when a constitutively active mutant of MAPK kinase 6 (MKK6), which selectively activates p38 MAPK, is co-expressed.

**Conclusions/Significance:**

Taken together, we here report p38 MAPK as a regulator of IL-24 expression and determine interference with destabilization mediated by the 3′ UTR of IL-24 mRNA as mode of action. As discussed in the present work these findings have important implications for our understanding of IL-24 as a tumor suppressor protein as well as an immune modulating cytokine.

## Introduction

The melanoma differentiation-associated gene-7 (mda-7) was discovered through subtraction hybridization of cDNA libraries prepared from melanoma cells [1]. The mda-7 gene was mapped to a gene cluster on chromosome 1q32 together with IL-10 cytokine family members, IL-10, IL-19, IL-20. As mda-7 shares considerable structural and sequence homology with IL-10 cytokine family members it was recognized as a member of this family and renamed interleukin-24 (IL-24) [2]. IL-24 signals through a heterodimeric receptor complex consisting of the IL-20Rβ subunit together with either the IL-20Rα subunit or the IL-22Rα subunit. The IL-20Rα/IL-20Rβ complex is shared with IL-19 and IL-20 whereas the IL-22Rα/IL-20Rβ complex is shared with only IL-20 [3], [4]. Binding of IL-24 as well as IL-19 and IL-20 to the receptor complexes results in signal transducer and activator of transcription 3 (STAT3) -activation [4].

In general, research on IL-24 can be divided into two categories: IL-24 as a classical cytokine with immune modulating properties or IL-24 as a protein with tumor suppressor effects.

Forced IL-24 expression driven by a replication incompetent adenoviral vector (ad.mda-7) has been demonstrated to selectively induce growth suppression and apoptosis in a broad spectrum of malignant cell lines *in vitro* while leaving normal cells unaffected [5], [6], [7], [8]. It appears that these effects are independent of the so far identified IL-24 receptor complexes and JAK/STAT-signaling [7]. Secreted IL-24 has been reported to be anti-angiogenic and to sensitize tumor cells to radiation therapy and chemotherapy [9]. Furthermore, intra-tumoral administration of ad.mda-7 in a phase I clinical trial showed evidence of clinical tumor suppressor effects [10], [11] and in support of the tumor suppressor effect of IL-24, there appears to be an inverse correlation between IL-24 expression levels and melanoma progression [5], [12].

Although the majority of reports focus on IL-24 as a tumor suppressor gene a few studies have investigated immune modulating properties of IL-24 and its potential role in inflammatory diseases [13], [14], [15]. In synovial fluid mononuclear cells, IL-24 induced secretion of mononuclear chemoattractant MCP-1 and in cultured PBMCs IL-24 induced expression of IL-6, tumor necrosis factor-α (TNF-α), and interferon-γ [14], [15]. Increased expression of IL-24 has been reported in affected joints of patients with rheumatoid arthritis and in lesional skin of patients with psoriasis [15], [16], [17]. Keratinocytes express both the IL-20Rα/IL-20Rβ complex and the IL-22Rα/IL-20Rβ complex and stimulation of normal human epidermal keratinocytes (NHEK) with IL-24 *in vitro* induced STAT3 activation [13], [18]. Furthermore, IL-24 stimulation of NHEK resulted in altered keratinocyte differentiation pattern, increased cell proliferation, and expression of a number of psoriasis-related genes [13]. Taken together these findings suggest a role for IL-24 in the pathogenesis of psoriasis and other inflammatory conditions.

Cellular sources of IL-24 include, monocytes, T-cells [19], NHEK [18], and melanocytes [5]. In peripheral blood mononuclear cells (PBMCs) IL-24 gene expression is induced by exposure to concanavalin a- [3], lipopolysaccharide (LPS)-, or cytokines [19] whereas in keratinocytes only IL-1β has been reported to induce IL-24 mRNA expression [18]. At the transcriptional level IL-24 expression has been reported to be regulated by the transcription factors activator protein-1 and CCAAT-enhancer-binding proteins [20]. On the other hand, studies in melanoma cells and PBMCs have suggested, that post-transcriptional gene regulation by mRNA stabilization is of major importance to IL-24 gene expression [19], [21]. This is further supported by the fact that the 3′ UTR of IL-24 mRNA contains mRNA-destabilizing AU-rich elements (ARE) [21]. Yet the mechanisms responsible for the regulation of IL-24 mRNA stability remain unidentified.

A study by Zhang *et al* reported ras oncogene-induced IL-24 expression indicating that IL-24 gene expression could be regulated by MAPK signaling[22]. The p38 MAPK is known to regulate the expression of cytokines through interfering with ARE-dependent destabilization of cytokine-mRNA [23], [24], [25], [26]. In a previous study we identified p38 MAPK as crucial for the expression of the IL-24-related cytokine, IL-20 [27]. As several of the stimuli reported to induce IL-24 mRNA expression (e.g. IL-1β in NHEK [18] and LPS in PBMCs [19]) are known p38 MAPK activators we examined the role of p38 MAPK in the regulation of IL-24 gene expression. In the present study we discover that IL-24 expression in NHEK is dependent on p38 MAPK activation, and we demonstrate for the first time that p38 MAPK regulates IL-24 gene expression at a posttranscriptional level by interfering with destabilization mediated by the 3′ UTR of IL-24 mRNA.

The findings of the present study have important implications for the understanding of IL-24 not only as an immune modulating cytokine but also as a tumor suppressor gene.

## Results

### p38 MAPK Activation in NHEK Treated with Anisomycin or IL-1β

**Figure 1:**
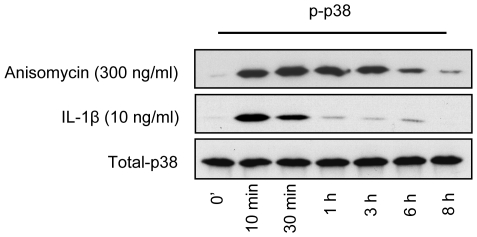
Time course studies of p38 MAPK phosphorylation in NHEK treated with anisomycin (300 ng/ml) and IL-1β (10 ng/ml). Western blotting of equivalent amounts of whole-cell protein extracts from NHEK treated with anisomycin or IL-1β for the indicated time periods. The proteins were separated by SDS-PAGE, blotted onto a nitrocellulose membrane and probed with an anti-phospho-p38 MAPK antibody. Equal loading was confirmed by incubating with anti-total-p38 MAPK. A representative gel of three different experiments is shown.

Time course studies of p38 MAPK phosphorylation in NHEK treated with anisomycin (300 ng/ml) or IL-1β (10 ng/ml) were performed to assess their p38 MAPK activating capabilities. Whole cell extracts were prepared and phosphorylation of p38 MAPK assessed by Western blotting (Figure 1). Stimulation for 10 minutes with anisomycin as well as IL-1β leads to increased p38 MAPK phosphorylation. In anisomycin-treated cells this increase was maintained for at least 6–8 hours whereas in IL-1β-stimulated cells p38 MAPK phosphorylation levels rapidly declined after 30 minutes. Thus, anisomycin stimulation of NHEK resulted in a more pronounced and sustained p38 MAPK phosphorylation than IL-1β stimulation as determined by Western blotting.

### IL-24 mRNA Expression Is Induced by Anisomycin and IL-1β in NHEK

**Figure 2:**
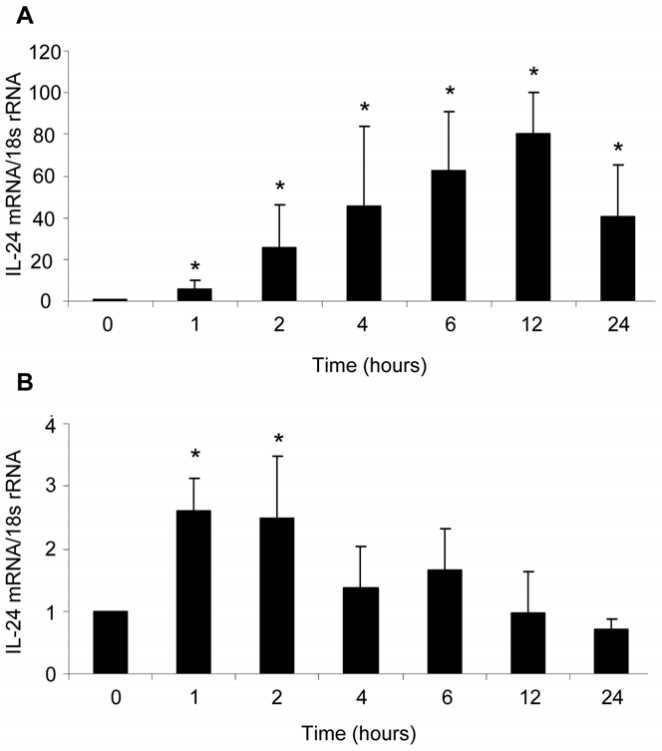
Time course studies of IL-24 mRNA expression. NHEK were incubated with (A) anisomycin (300 ng/ml) or (B) IL-1β (10 ng/ml) for the indicated time periods and IL-24 mRNA expression levels (relative to housekeeping gene 18s rRNA) were determined using quantitative RT-PCR. *p<0.05 compared with cells harvested before stimulation (0 hours) (A, n = 6 cultures, B, n = 5 cultures). Bars indicate mean ± SD.

Having established the p38 MAPK activation profile of anisomycin and IL-1β we wanted to study the dynamics of IL-24 mRNA expression in NHEK incubated with these two stimuli. Therefore, time course studies on IL-24 mRNA expression in NHEK stimulated with anisomycin (300 ng/ml) or IL-1β (10 ng/ml) were conducted (Figure 2). IL-24 mRNA expression levels were determined with quantitative reverse transcription polymerase chain reaction (qRT-PCR). In anisomycin treated cells IL-24 mRNA levels were increased after one hour reaching a maximal level of expression after 12 hours of stimulation. At this time point, IL-24 mRNA levels showed an 80-fold induction compared with non-stimulated cells (Figure 2A). Significantly increased levels of IL-24 mRNA levels were observed in IL-1β-stimulated NHEK after one and two hours (Figure 2B). Hereafter IL-24 mRNA levels steadily declined. Maximal IL-24 mRNA expression was detected after only 1 hour of IL-1β stimulation at which point IL-24 mRNA levels were 2.6 fold upregulated compared with cells not stimulated. Thus, the observed induction of IL-24 mRNA expression in NHEK treated with anisomycin or IL-1β corresponded to the p38 MAPK activation profile of these stimuli supporting a role for this kinase in the regulation of IL-24 expression.

### The Dynamics of IL-24 Protein Expression in NHEK Treated with Anisomycin

**Figure 3:**
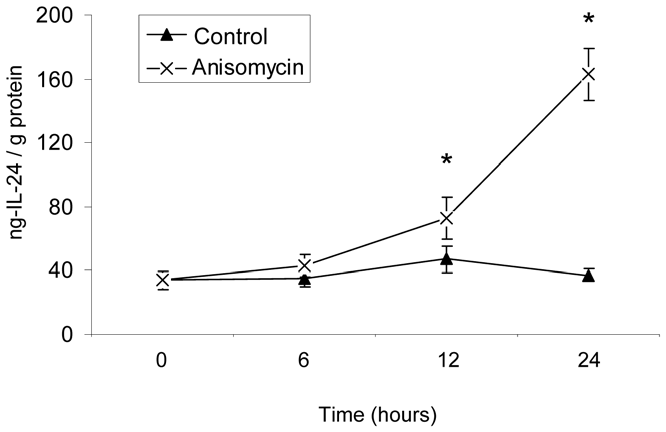
IL-24 protein expression in the supernatant of NHEK treated with medium control or anisomycin (300 ng/ml). NHEK were cultured in the presence or absence of anisomycin for up to 24 hours. IL-24 protein levels in the supernatant were analyzed by ELISA. IL-24 protein concentrations were normalized to the calculated total protein concentration of the whole cell extracts.*p<0.05 compared with cells harvested before stimulation (0 hours) (n = 5 cultures). Points indicate mean ± SEM.

To study the kinetics of IL-24 protein expression, NHEK were incubated with anisomycin (300 ng/ml) for 0, 6, 12, and 24 hours. IL-24 protein levels were subsequently analyzed in whole cell extracts as well as the supernatant using enzyme-linked immunosorbent assay (ELISA) (Figure 3). Consistently, intracellular IL-24 protein levels remained below the detection limit of the IL-24 ELISA assay (data not shown). In the supernatant of medium-incubated keratinocytes IL-24 protein remained constantly at a low level whereas increased levels of IL-24 protein were detected in the supernatant of anisomycin-stimulated NHEK at 12 and 24 hours. The increased IL-24 mRNA expression in anisomycin-stimulated NHEK therefore, leads to an increased IL-24 protein expression. The newly synthesized protein is not stored inside the cell but immediately secreted.

### p38 MAPK Is a Regulator of IL-24 Expression

**Figure 4:**
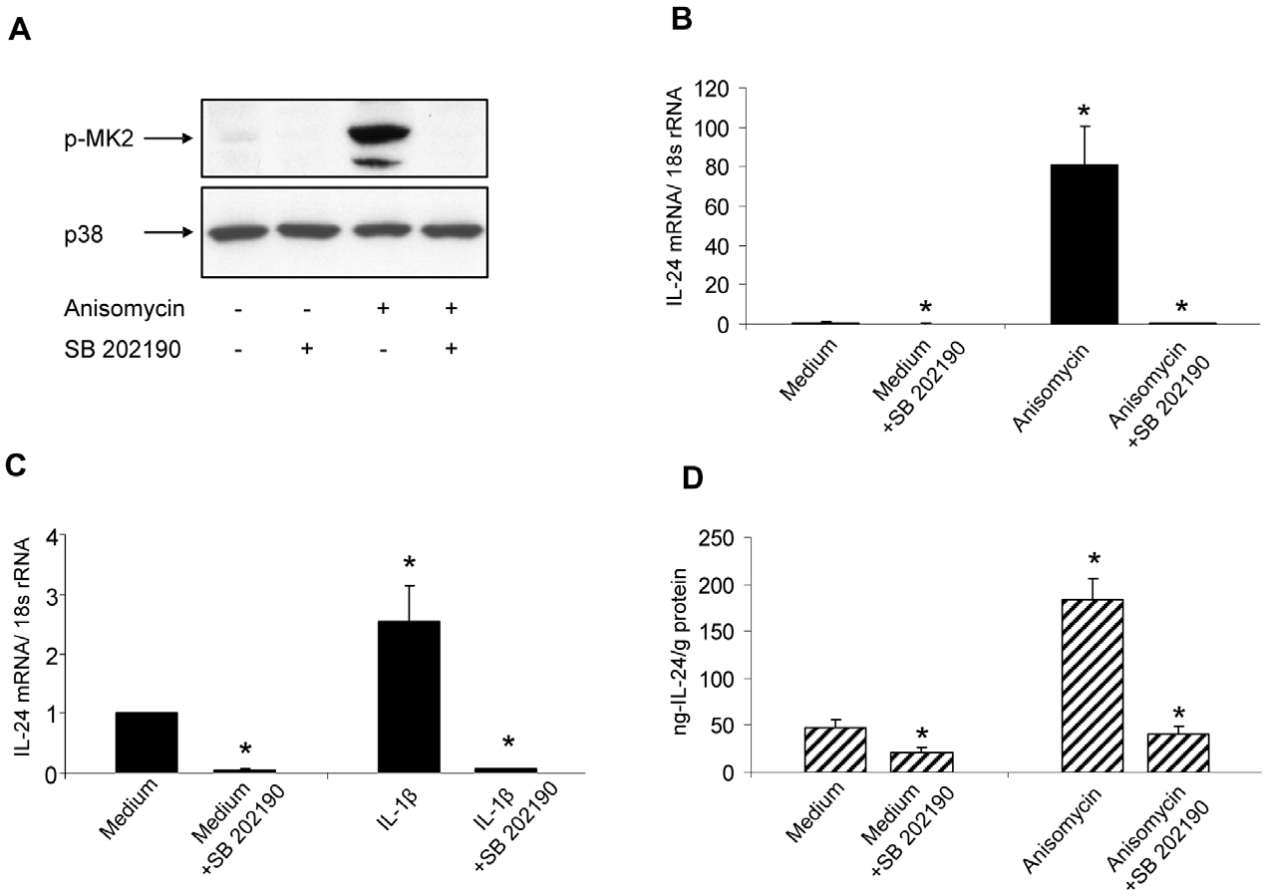
Inhibition of p38 MAPK activation abrogates IL-24 expression. (A) Whole cell extracts were prepared from NHEK incubated with or without (control) anisomycin (300 ng/ml) for 30 minutes in the absence or presence of the 38 MAPK inhibitor SB 202190 (10 µM). Proteins were separated by SDS-PAGE. After electroblotting, separated proteins were probed with anti-phospho-MK2 (recognizing the phosphorylated form of MK2 isoforms 1 and 2) and anti-total p38 MAPK. A gel representative of three different experiments is shown. NHEK were cultured with or without SB 202190 (10 µM) for 30 minutes before incubation with (B) anisomycin (300 ng/ml) or medium control for 4 hours or (C) IL-1β (10 ng/ml) or medium control for 2 hours. Total RNA was extracted and IL-24 mRNA (relative to housekeeping gene 18 s RNA) levels were determined. *p<0.05 compared with medium control (n = 5 cultures). Bars indicate mean ± SD. (D) NHEK were cultured in the presence or absence of SB 202190 (10 µM) with medium control or anisomycin (300 ng/ml) for 18 hours. Using ELISA IL-24 concentration in the supernatants was determined and subsequently normalized to the total protein concentration of whole cell extracts. *p<0.05 compared with medium control (n = 5 cultures). Bars indicate mean ± SD.

To further investigate the role of p38 MAPK in IL-24 expression we used the p38 MAPK inhibitor SB 202190. First, we investigated the efficacy of this inhibitor in disrupting p38 MAPK activity by preincubating NHEK with SB 202190 (10 µM) before treatment with medium control or anisomycin (300 ng/ml) for 30 minutes. For the assessment of p38 MAPK activation, the phosphorylation of p38 MAPK-substrate mitogen activated protein kinase-activated protein kinase 2 (MK2) was analyzed by Western blotting (Figure 4A). Preincubation with SB 202190 resulted in complete abrogation of MK2-phosphorylation reflecting complete disruption of p38 MAPK signaling by the inhibitor.

Next, we wanted to determine the impact of disrupted p38 MAPK activation on anisomycin- and IL-1β- induced IL-24 mRNA expression. Therefore, NHEK were incubated with or without SB 202190 (10 µM) before treatment with medium or anisomycin (300 ng/ml) for 6 hours (Figure 4B) or medium or IL-1β for 1 hour (Figure 4C), respectively. Inhibition of p38 MAPK before stimulation with anisomycin or IL-1β downregulated IL-24 mRNA expression to a level even below that observed in medium treated NHEK. In a separate set of experiments the effect of SB 202190 on anisomycin-induced IL-24 protein expression was investigated (Figure 4D), and in accordance with what we observed at the mRNA-level, IL-24 protein concentration in the supernatant of SB 202190-preincubated anisomycin-stimulated-NHEK was even below what was observed in the supernatant of NHEK incubated only with medium.

### The p38 MAPK Regulates IL-24 mRNA Stability

**Figure 5:**
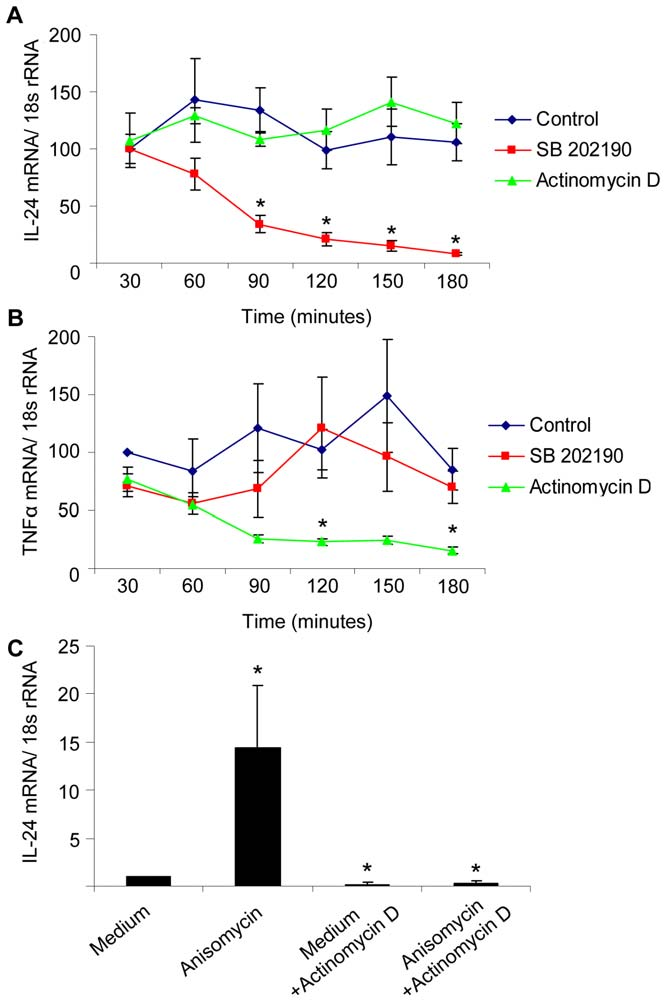
p38 MAPK regulates IL-24 expression at a posttranscriptional level. (A and B) NHEK were pre-stimulated with anisomycin (300 ng/ml) for 2 hours at which point medium (control), the transcription inhibitor actinomycin D, or SB 202190 were added to the cultures. Cells were then harvested at 30 minute intervals, total RNA extracted and (A) IL-24 and (B) TNFα-mRNA levels (relative to 18 s RNA) were determined. *p<0.05 compared with control (n = 5). Points indicate mean ± SD. (C) As control NHEK were pre-incubated with or without actinomycin D for one hour before treatment with medium or anisomycin (300 ng/ml) for another 2 hours. Total RNA was extracted IL-24 mRNA levels (relative to 18 s RNA) were determined. *p<0.05 compared with medium control (n = 4). Bars indicate mean ± SD.

Having discovered p38 MAPK as an important player in the regulation of IL-24 expression we wanted to investigate the underlying mechanisms. Therefore, we analyzed the impact of p38 MAPK inhibition on IL-24 mRNA stability. First, NHEK were incubated with anisomycin (300 ng/ml) for two hours, thus allowing activation of p38 MAPK and induction of IL-24 mRNA expression. Then cells were added either medium (control), the inhibitor of transcription actinomycin D (7.5 µg/ml) or the p38 MAPK inhibitor, SB 202190 (10 µM). Cells were subsequently harvested at 30 minutes intervals for up to 180 minutes and IL-24 mRNA expression determined (Figure 5A). Inhibition of transcription with actinomycin D did not lead to a downregulation of IL-24 mRNA levels throughout the 180 minutes of the study suggesting that IL-24 mRNA is relatively stable in anisomycin-stimulated NHEK. However, inhibition of p38 MAPK with SB 202190 resulted in a significant downregulation of IL-24 mRNA after 90 minutes (34 fold vs 134 fold in control). Simultaneous inhibition of transcription (actinomycin D) and the p38 MAPK (SB 202190) resulted in a downregulation of IL-24 mRNA expression comparable to that achieved by SB 202190 alone (data not shown). Hence, p38 MAPK appears to be essential for IL-24 mRNA stability. As an internal assay control TNF-α mRNA expression was also determined. Incubation with SB 202190 did not affect TNF-α mRNA expression levels significantly whereas degradation of TNF-α mRNA could be detected after 120 minutes in cells incubated with actinomycin D (Figure 5B).

As control NHEK were also incubated with actinomycin D (7.5 µg/ml) for one hour before stimulation with anisomycin (300 ng/ml) for 2 hours (Figure 5C). In this setup preincubation with actinomycin D before treatment with anisomycin downregulated IL-24 mRNA levels to 0.35 fold compared with 14.5 fold in anisomycin-stimulated NHEK not preincubated with actinomycin D. Thus, as transcription was blocked before anisomycin stimulation IL-24 mRNA rapidly decayed reflecting a short half-life of IL-24 mRNA in cells where p38 MAPK activation has not been induced.

### The 3′ UTR of IL-24 mRNA Mediates Rapid Degradation and p38 MAPK-Induced Stabilization

**Figure 6:**
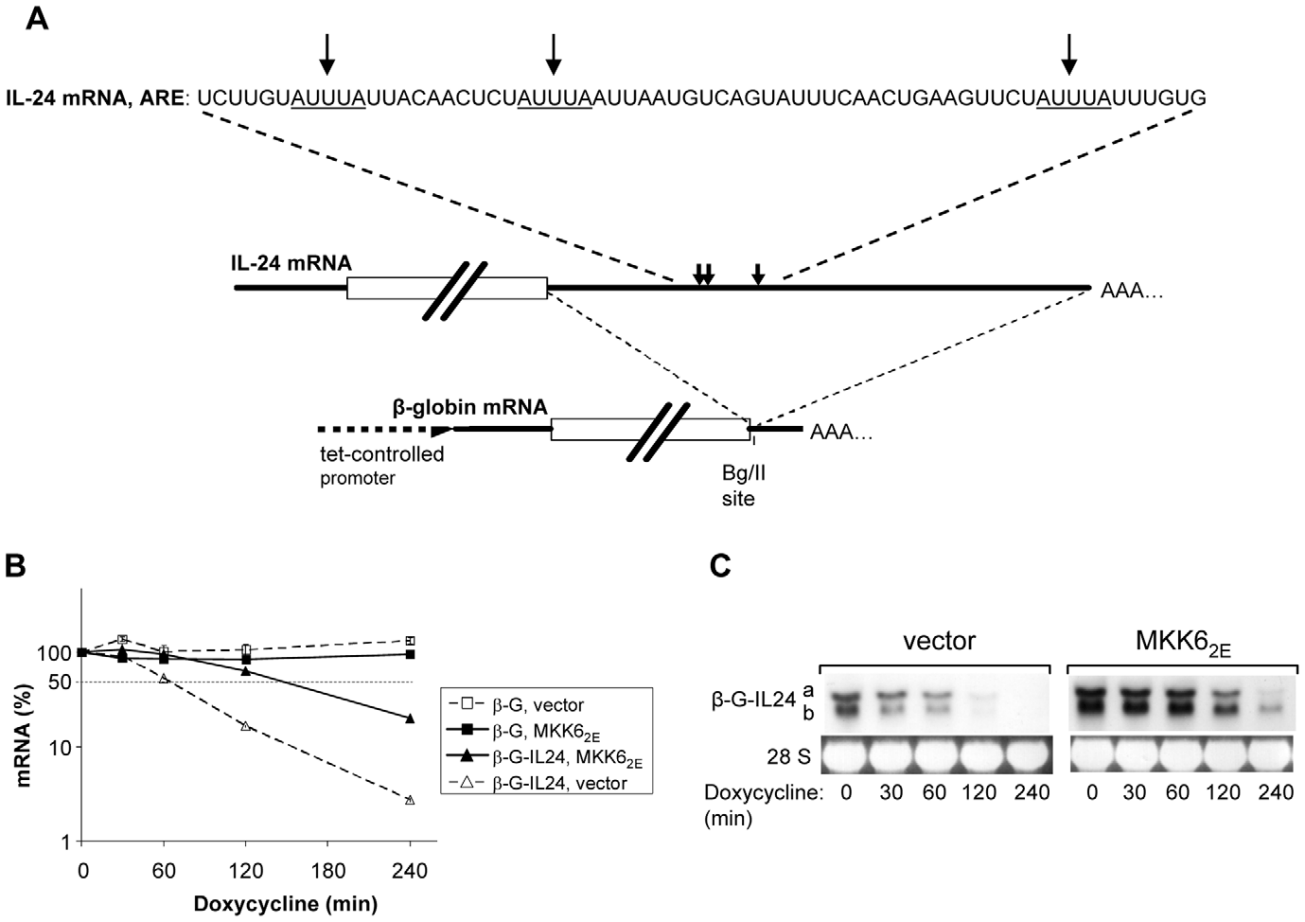
The 3′ UTR of IL-24 mRNA mediates rapid degradation and p38 MAPK-induced stabilization of a reporter mRNA. (A) Scheme of IL-24 mRNA and insertion of its 3′ UTR into *tet-off* vector-expressed β-globin mRNA. The three AUUUA-motifs (indicated by arrows) are displayed. (B) HeLa cells were transfected with plasmids expressing the β-globin mRNA without insertion (β-G) or with the IL-24 3′ UTR inserted (β-G-IL24) and with empty vector or a plasmid expressing constitutively active MKK6 (MKK6_2E_). Total RNA was isolated at the indicated times after stopping transcription with doxycycline (3 µg/ml). mRNA amounts were determined by qRT-PCR (means ± SD of triplicates) and expressed relative to the amount at the time of doxycycline addition ( = 100%). (C) mRNAs from a separate experiment carried out as described for (B) were analyzed by Northern blot with a β-globin antisense probe. Two species of the hybrid mRNA (β-G-IL24 a and b) were detected. Ethidium bromide staining of 28 S rRNA is shown as a loading control. Similar results were obtained in two separate experiments.

p38 MAPK stabilizes mRNAs by interfering with rapid degradation imposed by AU-rich elements in their 3′ UTR. The IL-24 3′ UTR contains three AUUUA motifs typical for AU-rich elements (Figure 6A). To test if indeed the IL-24 3′ UTR harbors the elements mediating rapid degradation and p38 MAPK-dependent stabilization, a *tet-off* reporter plasmid was constructed. The plasmid expresses the stable β-globin mRNA (t/2>10 h, not shown) with the IL-24 3′ UTR inserted downstream of the stop codon (Figure 6A). The kinetics of degradation of the chimeric mRNA and of the β-globin mRNA without insertion was determined in HeLa cells by qRT-PCR of RNA isolated at different time points following inhibition of transcription by doxycycline. The half life of the chimeric mRNA was also determined by Northern blot (Figure 6C). Two mRNA species were detected, probably reflecting two different polyadenylation sites. Both species are rapidly degraded, demonstrating that the IL-24 3′ UTR exerts effective destabilization.

The effect of p38 MAPK activation on degradation of the chimeric mRNA was assayed by co-expressing a constitutively active mutant of the MAPK kinase MKK6 (MKK6_2E_). MKK6 selectively activates p38 MAPK [28] and has been used in the past to show p38 MAPK-dependent stabilization of several mRNAs containing AU-rich elements [24], [25]. MKK6_2E_ co-expression caused a marked stabilization of the β-globin-IL-24 chimeric mRNA. Hence, we conclude that rapid degradation of IL-24 mRNA under basal conditions and stabilization by p38 MAPK are dependent on the 3′ UTR of IL-24.

## Discussion

In the present study we investigated the ability of anisomycin and IL-1β to induce p38 MAPK mediated IL-24 expression. Anisomycin is a specific p38 MAPK and JNK activator [29] whereas IL-1β in addition to p38 MAPK and JNK also activates several other signaling pathways including ERK1 and 2 [30], and IKK-β [27]. We discovered a correlation between the level of p38 MAPK activation and IL-24 mRNA expression. Inhibition of p38 MAPK activation with the p38 MAPK inhibitor SB 202190 blocked IL-24 mRNA and protein expression in both stimulated and un-stimulated cells, demonstrating the importancy of this kinase in IL-24 expression. These findings were also confirmed by separate sets of experiments where NHEK were stimulated with 12-O-Tetradecanoylphorbol-13-acetate (data not shown).

In accordance with what has been observed in LPS-treated PBMCs [19] we found IL-24 mRNA to be a relatively stable molecule in keratinocytes stimulated with anisomycin. However, when p38 MAPK was inactivated with SB 202190 the IL-24 mRNA half-life was significantly reduced reflecting increased mRNA instability and an accelerated IL-24 mRNA decay. Hence, our results suggested p38 MAPK to regulate IL-24 expression by mRNA-stabilizing mechanisms. It is a well established fact that p38 MAPK control expression of cytokines, growth factors or proto-oncogenes through regulation of mRNA decay [23]. The p38 MAPK controls the binding affinity of ARE-binding proteins either directly or through activation of downstream kinases (e.g. MK2, MK3 and the MAPK-interacting kinases) [25], [26]. Altered binding of this class of proteins to ARE in the 3′ UTR of mRNA ultimately leads to increased stability of a specific mRNA and expression of its encoded protein [31]. In keeping with a previous study on melanoma cells [21], insertion of the 3′ UTR of IL-24 mRNA induced degradation of the β-globin reporter mRNA in our assay. Selective activation of p38 MAPK by expressing a constitutively active form of the p38-selective MAPK kinase 6 (MKK6) resulted in increased stability of the IL-24 3′ UTR-β-globin reporter mRNA assay. Taken together these findings demonstrate that p38 MAPK regulates IL-24 mRNA expression by interfering with destabilization exerted by the 3′ UTR of IL-24 mRNA.

IL-24 expression can be detected in melanocytes and early melanomas but expression levels decrease as melanomas advance becoming virtually undetectable in metastatic disease [5], [12]. Furthermore, induction of terminal differentiation in melanoma cells with fibroblast interferon and mezerein lead to an increase in IL-24 mRNA expression primarily through an increased stability of IL-24 mRNA [1], [21]. p38 MAPK is known to be involved in the growth and differentiation of normal cells [32]. In melanoma cells p38 MAPK has been reported to mediate α-melanocyte stimulating hormone-induced differentiation [33], [34]. Thus, the gradual loss of IL-24 expression as melanocytes transform into aggressive melanoma cells and the regained expression of IL-24 in terminally differentiated melanoma cells may well be explained by differentiation-associated alterations in the activity of p38 MAPK, its downstream kinases and RNA-binding proteins.

Intriguingly, IL-24 has been demonstrated to exert its effects in malignant cells by inducing cellular stress leading to p38 MAPK activation [8], [35], [36]. IL-24-induced p38 MAPK activation promoted survival of chronic lymphocytic leukaemia B-cells [36] whereas ad.mda-7-induced p38 MAPK activation in melanoma cells lead to apoptosis [8]. These findings correspond with the fact that p38 MAPK primarily is considered a tumor suppressor pathway although in certain settings it may also promote the opposite [37]. Recently, IL-24 was reported to induce its own expression in cancer cells through mRNA stabilization [38]. It was suggested that IL-24 through a positive autocrine loop induced amplification of its own expression as well as cellular stress leading to apoptosis of malignant cells [38]. Our findings complete this loop: Hence, forced expression with ad.mda-7 in malignant cells leads to cellular stress and p38 MAPK activation which in turn stabilizes endogenous IL-24 mRNA further enhancing IL-24 expression and cellular stress eventually resulting in apoptosis.

p38 MAPK is assumed to be of major importance in the inflammatory response and increased activation of this pathway and its downstream kinases has been reported in inflammatory conditions such as psoriasis [39], [40]. In accordance, the expression of several cytokines assumed to play a role in the pathogenesis of psoriasis (i.e. IL-20[27], TNF, IL-8, and IL-6) are p38 MAPK-regulated. Thus, the increased expression of IL-24 mRNA in lesional psoriatic skin [16], [17] corresponds nicely with the increased activation of p38 MAPK in psoriasis and indicates a role for IL-24 in the pathogenesis of psoriasis.

However, it appears that many of the characteristics of IL-24 are shared with other members of the IL-10 family, in particular IL-20. For instance, IL-20 and IL-24 share receptor complexes, have considerable sequence homology, activate STAT3, and have similar effects on keratinocytes (induces proliferation, alter differentiation pattern and expression of overlapping sets of genes). IL-20 and IL-24 expression are induced by the same stimuli in identical cell types, i.e. IL-1β in keratinocytes [18] and LPS in monocytes [19]. Furthermore, IL-20 mRNA also contains several AREs in its 3′ UTR [41], and IL-20 expression is also strongly dependent on p38 MAPK activation [27]. Altogether, these findings point to redundancy among the IL-10 family members, which must be considered when designing drugs targeting these cytokines for the treatment of inflammatory conditions. In case of explicit redundancy within the IL-10 cytokine family, modulation of their expression could prove more efficacious than targeting the cytokines individually. Our findings point to p38 MAPK and its downstream kinases as potential candidates for this.

In summary, we identify p38 MAPK as a regulator of IL-24 expression and determine interference with destabilization mediated by the 3′ UTR of IL-24 mRNA as mode of action. These findings help us to understand the altered expression pattern of IL-24 in malignant tissues, and they provide new important knowledge if IL-10 cytokine family members are to be targeted in the treatment of inflammatory conditions such as psoriasis and rheumatoid arthritis.

## Materials and Methods

### Keratinocyte Cultures

NHEK were obtained by trypsinization of skin removed from patients undergoing plastic surgery [42]. First passage keratinocytes were grown in serum-free keratinocyte growth media with human recombinant epidermal growth factor, bovine pituitary extract and gentamicin (Gibco/Invitrogen, Carlsbad, CA). Fresh media was added every second day and cells were grown to approximately 60% confluency. At this point the growth factor supplemented media was changed to non-growth factor supplemented media. After another 24 hours the cells were stimulated. Anisomycin was purchased from Sigma-Aldrich (St. Louis, MO) and IL-1β was from RnD Systems (Abingdon, UK). As medium control sterile phosphate-buffered saline (Gibco/Invitrogen, Carlsbad, CA) with 0.15% bovine serum albumin was used. The keratinocytes were grown in 6-well plates (TPP, Trasadingen, Switzerland) at 37°C and 5% CO_2_. In selected experiments cells were incubated with inhibitor of the p38 MAPK pathway, SB 202190 (Calbiochem, San Diego, CA). SB 202190 was added 30 minutes prior to stimulation of the keratinocytes. Inhibition of transcription was achieved by incubating cells with actinomycin D (Calbiochem, San Diego, CA) 60 minutes prior to stimulation.

This study was conducted in accordance with the Helsinki Declaration principles. The medical ethical committee of Aarhus University Hospital approved the study. Informed, written consent was obtained from each patient.

### RNA Purification

The cell supernatant was discarded and while on ice the keratinocytes were washed once in ice-cold sterile phosphate-buffered saline (Gibco/Invitrogen, Carlsbad, CA). RNA was purified with SV Total RNA Isolation System (Promega, Madison, WI). Briefly; 175 µl of SV RNA Lysis Buffer was added and lysates prepared by manually scraping the bottom of the wells. 350 µl of SV RNA Dilution Buffer was added and tubes inverted 10 times to mix. Samples were then put on a heating block for three minutes at 70°C and afterwards centrifuged at 13000 g, at room temperature for 10 minutes. Then the supernatant was transferred to a new set of sterile eppendorf tubes. 200 µl of 96% ethanol was added to the supernatant and mixing was done by pipetting. The mixture was then transferred to spin baskets attached to a Vac-Man with Mini-Prep Vacuum Adapters (Promega, Madison, WI). Total RNA was purified according to the vacuum protocol, as described by the manufacturer. Ultimately, RNA was dissolved in RNase/DNase free water and stored at −80°C until further use.

### Quantitative RT-PCR

For reverse transcription, Taqman Reverse Transcription reagents (Applied Biosystems, Foster City, CA) were used according to manufacturer's instructions using random hexamers as primers. Reverse transcription thermal cycling was performed on a Peltier Thermal Cycler-200 (MJ Research, Inc. Waltham, MA). Conditions were a 25°C incubation step for 10 minutes, followed by a 48°C reverse transcription step for 30 minutes and finally a 95°C reverse transcriptase inactivation step for 5 minutes. cDNA was stored at −80°C. Primers and probes were purchased from Applied Biosystems (Foster City, CA). IL-24, IL-20, and TNF-α mRNA expression was determined with a Taqman 20X Assays-On-Demand (FAM-labelled MGB-probes) gene expression assay mix (assay ID: IL-24: Hs01114274_m1; IL-20: Hs00218888_m1; TNFα: Hs00174128_m1). The housekeeping gene used for normalization was 18s rRNA (assay ID: Hs99999901_s1). The expression of each gene was analyzed in triplicates. Reaction volume was 25 µl, consisting of 11.25 µl cDNA diluted in RNasefree-water, 1.25 µl 20X primer/probe mix and 12.5 µl Taqman 2X Universal PCR Master Mix (Applied Biosystems, Foster City, CA). PCR conditions were 2 minutes at 50°C, 10 minutes at 95°C followed by 50 cycles of 15 seconds at 95°C and 60 seconds at 60°C. Real-time PCR machine was a Rotorgene-3000 (Corbett Research, Sydney, Aus). Relative gene expression levels were determined by using the relative standard curve method as previously described [43].

### Whole Cell Extracts for ELISA

While the cells were on ice, the supernatant was transferred to sterile eppendorf tubes and snap frozen in liquid nitrogen. The keratinocytes were washed twice in ice-cold sterile phosphate-buffered saline and snap frozen in liquid nitrogen. Supernatant and cells were stored at −80°C until further use. Whole cell extracts were prepared from the keratinocytes. The six-wells were placed on ice and 120 µl cell lysis buffer (20 mM Tris-Base pH 7.5, 150 nM NaCl, 1 nM EDTA, 1 mM EGTA, 1% Triton X-100, 2.5 mM sodium pyrophosphate, 1 mM β-Glycerolphosphate, 1 mM sodium orthovanadate, 1 mM PMSF and 1× Complete protease inhibitor cocktail (Roche, Mannheim, Germany) were added to each well. The bottom of the wells was manually scraped with a rubber policeman and the samples were transferred to sterile eppendorf tubes. The samples were then sonicated and centrifuged at 10000 g for 10 minutes at 4°C after which the supernatant constitutes the cell lysate. Protein concentrations were determined with Bradford analysis using albumin for the standard curve. Samples were stored at −80°C until ELISA analysis.

### IL-24 ELISA

To quantify the concentration of IL-24 the human IL-24 DuoSet ELISA Development kit from R&D Systems, Abingdon, UK, was used according to the manufacturerer's instructions. Briefly; Maxisorp 96 well flat-bottom plates (NUNC, Roskilde, Denmark) were coated with 100 µl of capture antibody and incubated overnight at room temperature. Plates were then blocked with 300 µl of reagent diluent (1% BSA in PBS) and incubated for 1 hour at room temperature. Triplicates of 100 µl of samples and standards were added and plates were incubated for 2 hours at room temperature. A seven point standard curve using 2-fold serial dilutions and a high standard of 4000 pg/ml was used. Plates were then incubated with 100 µl of Detection Antibody for 2 hours at room temperature. 100 µl of Streptavidin-HRP was added and plates were incubated for 20 minutes at room temperature in the dark. Plates were then incubated with 100 µl of Substrate Solution in the dark at room temperature for 20 minutes. 50 µl of stop solution was added and the optical density was determined at 450 nm with a reference reading at 630 nm. All steps were followed by washing plates three times with wash buffer.

### Western Blot Analysis

Cells were washed in ice cold phosphate-buffered saline and lysed in a buffer containing 50 mM Tris-HCL, pH 6.8, 10 mM ditiothretiol, 10 mM β-glycerophosphate, 10 mM sodium fluoride, 0.1 mM sodium orthovanadate, 10% glycerol, 2.5% SDS added PMSF and a cocktail of proteinase inhibitors (Roche, Mannheim, Germany). The lysates were boiled for 3 minutes and while on ice incubated with benzon nuclease for 30 minutes and finally centrifuged for 13600 g for 3 minutes. The supernatant was transferred to a fresh eppendorf tube and stored at −80C° until further use. Protein concentrations were determined with Bradford analysis using albumin for the standard curve and equal amounts of whole cell extracts were separated with SDS-PAGE and blotted onto nitrocellulose membranes. Membranes were incubated with primary antibodies and detected by horseradish peroxidase-conjugated secondary antibodies in a standard ECL reaction (GE Healthcare, Wessling, Germany). Primary anti-phospho-p38 MAPK, -phospho-MK2 (Thr334), and -p38 MAPK antibodies, and secondary anti-rabbit antibody were from Cell Signaling Technology, Beverly, MA. A pre-stained marker (Invitrogen, Carlsbad, CA) and a biotinylated marker from Cell Signaling Technology were used for estimation of protein weight.

### Reporter Assays for mRNA Degradation

The 3′ UTR of IL-24 mRNA was analyzed for its effect on degradation of the stable β-globin mRNA following previously described protocols [31]. Briefly, a 3′ UTR fragment covering nt 895 to 1679 of human IL-24 mRNA (accession No. NM_006850) was obtained by RT-PCR with *Bgl*II-flanked primers (specific sequences: 5′ aatgtctagaccaggacct (sense), 5′ agcagggaatgtcatcaca (antisense), and cloned into the *Bgl*II-site of plasmid ptet-BBB, a *tet-off* vector which contains genomic sequences of rabbit β-globin [44]. HeLa cells constitutively expressing the tetracycline-controlled transactivator protein [45] were transfected by the calcium phosphate method, and the degradation kinetics of plasmid-expressed β-globin mRNA containing the IL-24 3′ UTR was determined using the tet-off system. Northern blot analysis of total RNA obtained after different times of inhibiting transcription by doxycycline was performed as described [23]. Results were quantified by a video imaging system and the TINA software for autoradiographs with suitable exposure times to avoid signal saturation. Samples of RNA were reverse-transcribed and real-time PCR was performed with a custom Taqman gene expression assay for rabbit β-globin mRNA (Applied Biosystems, Foster City, CA) according to the manufacturer's recommendations. Results were normalized to GAPDH mRNA (assay ID: Hs99999905_m1).

### Statistical Analysis

The significance of differences in figure 3, 5A, and 5B IL-24 mRNA levels and protein levels were determined by a paired t-test (two tailed). To test for normal distribution a probability test was made. In figure 2, 4 and 5c, 0h samples and medium control samples, respectively, was for every culture arbitrarily set to 1. All other samples were indicated as fold change compared with 0 h or medium control, respectively. For statistical analysis a two tailed one sample T-test (comparing the mean fold change with a hypothetical mean of 1) was used.
